# Deep Brain Stimulation and Brain–Spine Interface for Functional Restoration in Spinal Cord Injury

**DOI:** 10.3390/biomedicines13030631

**Published:** 2025-03-05

**Authors:** Barnabas T. Shiferaw, Max Y. Jin, Milan Patel, Lukas J. Henjum, Alaa Abd-Elsayed

**Affiliations:** Department of Anesthesiology, University of Wisconsin-Madison School of Medicine and Public Health, 600 Highland Avenue, B6/319 CSC, Madison, WI 53792, USA; bshiferaw@wisc.edu (B.T.S.); myjin@wisc.edu (M.Y.J.); mpatel0503@gmail.com (M.P.); lhenjum@wisc.edu (L.J.H.)

**Keywords:** spinal cord injury (SCI), neuromodulation, deep brain stimulation (DBS), brain–spine interface (BSI), epidural electrical stimulation (EES), neurorehabilitation

## Abstract

**Background/Objectives**: Spinal cord injury (SCI) presents significant challenges in restoring motor function, with limited therapeutic options available. Recent advancements in neuromodulation technologies, such as brain-spine interface (BSI), epidural electrical stimulation (EES), and deep brain stimulation (DBS), offer promising solutions. This review article explores the integration of these approaches, focusing on their potential to restore function in SCI patients. **Findings**: DBS has shown efficacy in SCI treatment with several stimulation sites identified, including the nucleus raphe magnus (NRM) and periaqueductal gray (PAG). However, transitioning from animal to human studies highlights challenges, including the technical risks of targeting the NRM in humans instead of rodent models. Additionally, several other regions have shown potential for motor rehabilitation, including the midbrain locomotor region (MLR) pathways, cuneiform nucleus (CnF), pedunculopontine nucleus (PPN), and lateral hypothalamic. DBS with EES further supports motor recovery in SCI; however, this approach requires high-DBS amplitude, serotonergic pharmacotherapy, and cortical activity decoding to attenuate stress-associated locomotion. BSI combined with EES has recently emerged as a promising novel therapy. Although human studies are limited, animal models have provided evidence supporting its potential. Despite these advancements, the effectiveness of DBS and combined systems remains limited in cases of complete central denervation. **Conclusions**: The integration and combination of DBS, BSI, and EES represent a transformational approach to treating and restoring function in patients with SCI. While further research is needed to optimize these strategies, these advancements hold immense potential for improving the quality of life in SCI patients and advancing the field of neuromodulation.

## 1. Introduction

Paraparesis is one of the most debilitating conditions worldwide and can be caused by a variety of etiologies. Complete or incomplete spinal cord injury (SCI) is one of the leading causes of paraparesis. Other causes include stroke, multiple sclerosis, and cerebral palsy. There are over five million Americans who live with partial or complete paralysis, the majority of whom are under the age of 65 [1].

Due to the quality of life and economic consequences associated with paraparesis, finding an effective treatment that can restore the function of paralyzed limbs is essential. Current management includes physical therapy, occupational therapy, pharmacological therapy, mobility assistive devices, transcranial magnetic stimulation, and others [2,3]. While these methods may provide some benefit, their efficacy in restoring function may be limited to somatic symptoms [4]. With no current consensus on how to restore function in patients with paraparesis, deep brain stimulation (DBS) has emerged as a possible treatment modality.

The application of DBS involves implanting electrodes into target regions of the brain [5]. The electrodes are connected to an implantable pulse generator controlled wirelessly by the physician to optimize frequency, pulse width, and voltage for a patient [5,6,7]. The technology used in DBS essentially adapts to that used in cardiac pacemakers and spinal cord stimulators [6]. DBS has been studied since the late 1800s, but the first commercially available device was not released until the mid-1970s [7]. In the early stages, DBS was often used in the treatment of Parkinson’s disease; however, indications have continued to expand. The FDA approves DBS for managing Parkinson’s disease and essential tremors [8].

Additionally, DBS has received FDA approval for the treatment of dystonia and obsessive–compulsive disorder. DBS has also been examined for use in treating chronic pain, Tourette’s, epilepsy, and depression [7]. The exact mechanism of action of DBS is yet not fully understood. Still, it is thought to work via the inhibition or excitation of neurons near the stimulator to modify neuronal activity [7].

In this narrative review, we examine evidence obtained from animal and human studies regarding the utility of DBS in incomplete SCI for functional restoration. Additionally, we explore the emergence of combined neuromodulation systems, including brain–spine interface (BSI) programs that interpret motor-evoking signals in the cortex and communicate with electrodes for spinal cord stimulation (SCS) or peripheral nerve stimulation (PNS). This novel combination therapy could use DBS as a modality to translate thoughts and transmit signals to other stimulators to perform movement in the previously paralyzed limb.

## 2. Methodology

The narrative explores and analyzes the current literature regarding deep brain stimulation and brain–spine interface models for functional restoration following spinal cord injury. The authors conducted a literature search through PubMed to identify all the relevant articles published before 15 December 2024. This review includes systematic, narrative, and human and animal studies. The inclusion criteria included articles discussing deep brain stimulation and brain–spine interface in patients or animals with spinal cord injury. The bibliographies of relevant articles were reviewed for additional sources. Articles were excluded if they were not in English or if the article’s primary aim was not related to the criteria previously outlined.

## 3. Findings

### 3.1. Deep Brain Stimulation for Functional Restoration Following SCI

With the inactivation of neurons being a primary concern for the lack of motor regeneration following SCI, DBS has been seen as a promising approach for developing functional restoration. DBS had its origin in neurological conditions, beginning in the treatment and restoration of movement in Parkinson’s disease and chronic pain [8]. Over time, the role of DBS has expanded into functional restoration and has a promising and unique approach to restoring function following SCI [9].

### 3.2. Deep Brain Stimulation for Functional Restoration Following SCI in Animal Studies

Previous animal studies demonstrated the potential role of DBS in improving function following SCI. A few foundational studies have paved the way for investigating key pathways in functional restoration following SCI.

One key study by Hentall et al. in 2012 investigated the stimulation of the nucleus raphe magnus (NRM) in the medial medulla or periaqueductal gray (PAG), which is located in the midbrain and has significant input into the NRM for the restorative function of motor activity in rats with SCI. The stimulation was administered between 0600 (6:00 a.m.) and 1800 (6:00 p.m.). The stimulation was given with 5 min trains alternated with 5 min of rest. The trains comprised 8 Hz, 1 ms, and 30 µA cathodal constant-current pulses as described by Hentall et al. In this study, the stimulation of both NRM and PAG demonstrated increased motor performance and axon myelination in rats with incomplete SCI at 14 weeks post-injury, indicating a possible treatment for patients with SCI [10].

Additional studies investigated DBS of the mesencephalic locomotor region (MLR) [11]. The MLR is an area of the midbrain responsible for locomotion production with low electrical stimulation thresholds [11,12]. Neurons in the MLR communicate with the motor cortex and basal ganglia. Damage to this region results in gait disturbances, and the restoration of this pathway may be associated with functional restoration in individuals with SCI. The MLR comprises two key areas commonly implicated in motor activity: the cuneiform (CnF) and pedunculopontine nucleus (PPN) [11].

Studies conducted by Bachman et al., 2013, reported that DBS of the MLR in rats with incomplete SCI demonstrated functional restoration close to pre-lesional levels at 4 weeks following SCI. In rats with incomplete SCI, DBS of the MLR resulted in markedly increased hindlimb function that was nearly fully restorative for swimming. Furthermore, DBS of the MLR allowed rats to regain basic movements of otherwise paralyzed hindlimbs [11].

Additional studies have been added to the body of evidence supporting DBS of the MLR for restorative function following SCI. Wang et al. in 2020 demonstrated that DBS of the MLR following SCI results in increased motor function via increased synaptic plasticity from the tropomyosin-related kinase B pathway [13]. Furthermore, Hofer et al. in 2022 specifically targeted the CnF of the MLF in rats with incomplete SCI and demonstrated increased motor function following DBS of CnF [14]. Strong and emerging evidence in support of DBS in animal studies demonstrates promising therapy that may benefit patients with SCI.

### 3.3. Deep Brain Stimulation for Functional Restoration Following SCI in Human Studies

The evidence of DBS in treating SCI in humans is limited but promising. In 2024, Cho et al. investigated DBS of the lateral hypothalamus (DBS^LH^) in two participants with incomplete SCI. These two participants exhibited varying ambulation levels with assistive devices following standard rehabilitation protocols; however, they demonstrated notable gate deficits before DBS therapy. For the participants enrolled in this study, DBS^LH^ demonstrated both immediate and long-term benefits to the study participants [15].

Immediately after DBS activation, the participants demonstrated improved lower limb muscle activity, kinematics, and muscle endurance, correlated with reduced patient-reported perceived walking effort [15]. Long-term benefits were reported following three months of structured rehabilitation with gait training and DBS^LH^, and improvement in walking was demonstrated as measured by 10 min and 6 min walk tests and lower extremity motor scores. The improvements at three months were recorded while the DBS was turned off in the study participants. Additionally, the functional recovery observed in these patients continued after DBS^LH^ was turned off and was not associated with any adverse events (AEs), including vital signs, weight, or hormonal abnormalities [15].

Additionally, Stieglitz et al. are conducting an ongoing clinical trial investigating DBS of the MLR for improving gait in patients with incomplete SCI (NCT03053791) [16]. DBS is a promising therapy for patients with SCI. Combined with other interventional modalities, it may offer a synergistic and groundbreaking approach to improving function in these patients.

### 3.4. Deep Brain Stimulation and Spinal Cord Stimulation

The potential for added and synergistic functional restoration with the combination of DBS and SCS has been under investigation. SCS involves electrode implantation near the dorsal columns of the spinal cord to deliver electrical pulses at the dorsal root ganglion and has been shown to induce neuroplasticity and improve volitional motor function in SCI [17,18]. Although medical polytherapy has been widely studied, more research needs to be conducted on how combining neuromodulatory therapies can best be applied in functional restoration [17].

Electrical epidural stimulation (EES) is a type of SCS that effectively improves functional recovery following SCI [18]. Animal studies by Angelin et al. in 2024 have shown increased locomotor function and neural cell counts following EES in SCI. The rodents in this study were implanted with a paddle electrode and an internal pulse generator. Additionally, the instruments provided 30 Hz, 500 μs, and 110 mV pulse for 40 min daily throughout the 60-day experiment [18]. In human studies, EES has significantly improved participants’ ability to stand and walk following SCI [19,20]. However, the combination of DBS and EES and its potential synergistic effect is still under investigation.

A study by Bonizzato et al., 2021, investigated the combination of DBS and EES in the functional recovery of rats following SCI [21]. This study demonstrated that DBS in combination with EES had a synergistic effect on improving locomotion compared to DBS alone or EES alone in rats with SCI. A few caveats from this study were also noteworthy for future investigation. One takeaway is that the effect of DBS depended on the severity of SCI, as DBS was only found to show improvement when at least 20% of the white matter tracts were spared. This injury-dependent impact of DBS was observed for DBS alone and DBS in combination with EES.

Considering the potential of a combined system utilizing DBS and EES is essential. A rodent model study used MLR DBS and EES to evaluate functional recovery after severe contusion SCI. The results showed that high-intensity DBS activated spinal locomotor networks and induced significant stress responses. To address this, researchers linked MLR DBS to the intention of walking by decoding motor activity, as previously mentioned. This approach improved EES-evoked locomotion and reduced the stress response. However, it is essential to note that the modest improvement in walking observed with this combined system may not justify the complexity of the technological framework required to implement it [12].

### 3.5. Brain–Spine Interface and Spinal Cord Stimulation for Functional Restoration

A promising technological breakthrough is using a BSI to restore function and movement in previously paralyzed limbs in SCI. The setup of BSI involves the implantation of electrocorticography (ECoG) or other arrays over the sensorimotor cortex, which records motor-evoking brain activity. This is in conjunction with the SCS in the lumbar spine to communicate with the lower extremities [22]. BSI works through the encoding and translating of motor-evoking thoughts into a computer-based system that serves as a digital bridge to connect with an implanted spinal cord stimulator to generate motor responses in a previously paralyzed limb [22]. Functional imaging via computerized tomography and magnetoencephalography is performed to identify the cortices involved in the movement of limbs, and the BSI is later calibrated with the EES for functional stimulation [23].

A study conducted by Lorach et al. in 2023 has been foundational in understanding BSI and EES in treating SCI in humans. The patient enrolled was a 38-year-old male with chronic tetraplegia following the incomplete SCI of the cervical spine caused by a biking accident ten years before enrollment in this study. This study aimed to implement and investigate the ability to implant a BSI to convert cortical motor signaling and connect it wirelessly to EES to regain function in the lower extremities following his injury. This patient could stand and walk naturally in the outpatient setting using BSI and EES. The BSI was calibrated within minutes, providing immediate improvements in hip flexor control and enabling a fivefold increase in muscle activity compared to baseline without the BSI. The patient’s rehabilitation with DBS and EES allowed him to stand and walk on complex terrains and even overground with crutches when the BSI was turned off. This study played a key role in implementing the combination of DBS and EES via a wireless digital bridge connecting the motor cortex and spinal cord to restore functional movement in a chronic tetraplegic patient [23].

The work of Lorach et al., 2023, was preceded by several key studies that investigated BSI and EES in animal models. In 2018, Bonizzato et al. investigated BSI and EES for gait rehabilitation in rats with SCI [24]. In rats with paralyzed legs, BSI and EES enabled the ability to approach restorative locomotion and achieve complex tasks such as stair climbing. In addition to long-term benefits, BSI and EES received immediate benefits, such as the ability to walk overground and adjust their foot clearance to attempt walking up stairs [24]. This study was foundational for demonstrating the proof of concept for future studies. 

An additional study by Capogrosso et al., 2016, provided further evidence demonstrating the benefit of BSI and EES in gait rehabilitation following SCI in *rhesus* monkeys. In this study, a BSI was implanted in the motor cortex of *rhesus* monkeys along with SCS through EES. After the validation of the BSI system, the monkeys received a unilateral thoracic corticospinal tract lesion. These monkeys were then followed over time, and as early as six days following SCI, the BSI system allowed for the weight-bearing motion of the paralyzed leg on a treadmill and overground [25]. This study in non-human primates and similar evidence in rat models provided promising evidence for further investigation in human trials.

A study conducted by Semejima et al. in 2021 investigated BSI and EES decoding to restore functional movement in rats after a cervical SCI. After being trained on a level-pressing task, the rats underwent the implantation of microelectrode arrays in their sensorimotor cortex. After a one-week recovery period, the rats were subjected to a series of decoding to ensure optimal neural connection. Decoding is the process of translating electrical activity in the cortex into meaningful information and operative commands. These signals can be linked to an external device to elicit motion through decoding, such as SCS. The decoding technique can be modified and optimized through algorithms and feedback looks. After reliability and accuracy testing was concluded, the implementation of the BSI occurred. Lastly, the decoded signals were used to control epidural stimulation and test the effectiveness of the BSI in facilitating forelimb movements in the rats. It was concluded that decoding movement intention and forelimb function were improved. Semejima et al. demonstrated that BSI could effectively translate neural signals into motor commands to stimulate neural pathways and thus provide functional restoration in rats following SCI [26]. The stimulation settings of studies included in this review are summarized in Table 1.

## 4. Discussion

This review focused on DBS, BSI, and other neuromodulations in treating SCI. The studies in this review included animal models that demonstrated the proof of concepts and supportive literature along with evidence of benefit in several promising human studies.

In the context of DBS, emerging evidence supports the benefit of DBS alone in treating SCI. However, there is still some debate surrounding the optimal site and pathway for DBS, as different areas have shown significant benefits. DBS of the NRM and the PAG have both demonstrated their efficacy. However, when looking at the transition from animal to human studies, DBS of the PAG may be of particular interest. In humans, the NRM in the medial medulla presents a technically risky target for intervention and direct stimulation, so the activation of the PAG can offer translation information for DBS along this pathway [10]. Additionally, when investigating the MLR pathway, the CnF and PPN are areas of interest commonly implicated in the motor pathway [11,14]. In human studies, DBS of the lateral hypothalamus was performed and has shown immediate and long-term benefits, suggesting that there may be several areas of interest for DBS for motor rehabilitation [15].

Regarding DBS in combination with SCS, such as EES, evidence demonstrates its benefit for motor rehabilitation is SCI. However, several key considerations exist for implementing DBS and EES. DBS and EES alone were not effective in producing locomotion in rats with complete paralysis. They required serotonergic pharmacotherapy to enable weak but consistent locomotion before the effects of DBS and EES could be observed. Lastly, the combination of DBS and EES requires high amplitudes of DBS, which triggers locomotion associated with stress. DBS had to be linked to the intention to walk and decoded from cortical activity using a learning algorithm to suppress this stress response [21].

The key factors to consider when evaluating if MLR DBS with EES is a good option for post-SCI are the stimulation site, the variability in efficacy across species when using combinatorial strategies, and the differing outcomes associated with various electrode types. These factors will contribute to the overall view of how MLR DBS, in combination with EES, may be used as a neuromodulation technique for functional restoration, specifically with SCI [12,21].

BSI, combined with EES, shows promising evidence in motor and functional rehabilitation in treating SCI [22,23,25,26]. The novelty of connecting the motor cortex through a digital bridge to generate motor responses in previously paralyzed individuals has the potential to be a very promising therapy. Given that the initial stages of BSI implantation and mapping of the motor cortex can be an invasive process, it is not a light undertaking. Still, it has the potential to be very powerful. Several key studies in animal models have implemented a proof of concept; however, they are limited but promising.

### 4.1. Shortcomings of DBS and Combined Systems

Although DBS and combined DBS systems may have the potential for functional restoration in many patients, they may be ineffective in cases where complete denervation is the cause of paraparesis. Central denervation remains a permanent process with no proven therapies to induce regeneration [27]. PNS is the only type of neuromodulation for patients with peripheral denervation that has been evidenced to benefit nerve regeneration [28]. However, PNS is only effective in accelerating regeneration and cannot control the direction of new axons. Furthermore, peripheral nerve regeneration is time-dependent, so any therapy must be implemented within two weeks of injury for maximum benefit. Additionally, therapies involving DBS have varying treatment durations, and treatment is often complex and variable.

### 4.2. Complications of DBS and Combined Systems

As with any surgical procedure, there are risks of AEs associated with DBS. Most AEs are related to the device itself or the implantation procedure, with common ones being infection, explantation, lead fracture, and erosion [29]. Other non-device-related AEs include speech disturbance, weight gain, abnormal sensation, confusion, and depression. Although these AEs have been reported, their incidence remains low [29,30,31]. Interestingly, it has also been postulated that AEs vary by the stimulation target and settings. For example, DBS of the subthalamic nucleus may result in an increased risk of depression, and higher voltage stimulation may result in more severe depression [32]. SCS and PNS can cause similar device and implantation-related AEs, but non-device-related AEs are less frequently observed [19]. Common AEs include infection, implant site pain, and lead migration. While AEs may occur with SCS and PNS, they rarely cause serious or long-term damage.

### 4.3. Future Directions

Given the strong evidence for DBS in the treatment of SCI in animal models and the synergistic effects, continued studies investigating DBS in combination with other modalities, including EES or other forms of SCS, is an area of research that warrants further investigation. The limited evidence in human studies that showed immediate benefit following calibration and long-term benefit that was present even while the BSI was turned off shows a significant area of interest that warrants further research. Other neuromodulatory devices, such as PNS in combination with DBS or BSI, are also an area of study that should be investigated given their limited presence in the literature.

## 5. Conclusions

DBS is a promising therapy for functional restoration following SCI. The combination of DBS with other treatment modalities, such as EES, has been demonstrated to be a promising and effective therapy in animal and human studies. Also, BSI, in combination with EES, has shown benefits for motor rehabilitation, and further investigation of these systems and neuromodulation is warranted in human studies.

## Figures and Tables

**Table 1:** Study stimulation settings.

Study	Study Population	Level of Injury	Stimulation Target	Stimulation Details
DBS only				
Hentall and Gonzalez 2011 [10]	Rats	T8	Nucleus raphe magnus or periaqueductal gray	Monopolar leads Settings: 8 Hz, 1 ms, 30 µA Treatment length: 12 h daily, 5 min on/5 min off Mean of 3.4 days (range: 1–7, *n* = 13) 14 days (*n* = 19) Mean of 5.6 days (range: 4–7, *n* = 10)
Bachmann et al., 2013 [11]	Rats	T9	Mesencephalic locomotor region	Unipolar leads Settings: 50 Hz, 0.5 ms, 103.3 ± 71.0 μA Treatment length: Single stimulation session, duration NR
Wang et al., 2020 [13]	Rats	T10	Mesencephalic locomotor region	Unipolar leads Settings: 100 Hz, 0.5 ms, current NR Treatment length: 30 min daily for 4 weeks
Cho et al., 2024 [15]	Humans	T1 (*n* = 1) C5 (*n* = 1)	Lateral hypothalamus	Lead type NR Settings: 20 Hz, 60 µs, 2 mA (T1 patient), 40 Hz, 60 µs, 9–10 mA (C5 patient) Treatment length: 3 h per day (DBS “on” time NR), 3 times a week for 3 weeks in a rehabilitation program + 3 months independent use (DBS “on” time NR)
DBS + SCS				
Bonizzato et al., 2021 [21]	Rats	T8/T9	DBS: pedunculopontine nucleus (of the mesencephalic locomotor region) EES: L2 and S1 (dorsal column)	DBS: Monopolar leads Settings: 40 Hz, 200 µs, 50–250 μA EES: Monopolar leads Settings: 40 Hz, 0.2 ms, 50–350 µA Treatment length for both: 5 days per week, 30 min per day (totals weeks NR)
BSI + SCS				
Lorach et al., 2023 [23]	Human	C5/C6	Sensorimotor cortex; T11-L1	Settings: 40 Hz, 300 µs, 14–16 mA Treatment length: 40 sessions (1–3 h each) for 15 weeks in rehabilitation, currently in 3 year independent use phase
Bonizzato et al., 2018 [24]	Rats	T9–T10	Leg area of right motor cortex; L2 and S1	Settings: 40 Hz, 0.2 ms, 100–400 µA Treatment length: 5 days per week, 30 min per day
Capogrosso et al., 2016 [25]	*Rhesus* monkeys	T7–T8	Leg area of left motor cortex; T13–L1	Settings: 30–80 Hz, 1.5–3.9 V (other settings NR) Treatment length: 2 weeks (session details NR)
Samejima et al., 2021 [26]	Rats	C4	Rostral and caudal forelimb area of sensorimotor cortex; C5–C6	Settings: 50–100 Hz, 400 μs, 300 μA–1 mA Treatment length: 5 days per week, 5–25 min per day for 3–4 weeks

## Abbreviations

The following abbreviations are used in this manuscript:

SCI	spinal cord injury
DBS	deep brain stimulation
SCS	spinal cord stimulation
BSI	brain–spine interface
EES	Epidural Electrical Stimulation
NRM	nucleus raphe magnus
PAG	periaqueductal gray
MLR	midbrain locomotor region
CnF	cuneiform nucleus
PPN	pedunculopontine nucleus
PNS	peripheral nerve stimulation
DBS^LH^	deep brain stimulation of the lateral hypothalamus
AEs	adverse effects
